# A candidate competitive ELISA based on monoclonal antibody 3A8 for diagnosis of contagious bovine pleuropneumonia

**DOI:** 10.1007/s00253-024-13127-0

**Published:** 2024-04-08

**Authors:** Qi Wu, Zhixin Ma, Qiao Pan, Tong Liu, Yidan Zhang, Jiuqing Xin, Qingyuan Xu

**Affiliations:** 1 State Key Laboratory for Animal Disease Control and Prevention, Harbin Veterinary Research Institute, Chinese Academy of Agricultural Sciences, Harbin, China; 2 Institute of Western Agriculture, Chinese Academy of Agricultural Sciences, Xinjiang, China; 3 Shanghai Veterinary Research Institute, Chinese Academy of Agricultural Sciences, Shanghai, China

**Keywords:** Diagnostic marker, Contagious bovine pleuropneumonia, Monoclonal antibody, Competitive ELISA

## Abstract

**Abstract:**

For the development of a competitive ELISA (cELISA) to detect serum antibodies against the *Mycoplasma mycoides* subsp. *Mycoides* (*Mmm*) (strain PG1), the causative agent of contagious bovine pleuropneumonia (CBPP), all the proteins of this pathogen were analyzed. Then, a specific extracellular region of a transmembrane protein with the potential for diagnosis was identified. After that, a monoclonal antibody (Mab) named 3A8 was obtained using this extracellular region as an immunogen. Finally, a cELISA was established with the extracellular domain of this transmembrane protein as the coating antigen, Mab 3A8 as the competitive antibody, and HRP-labeled goat anti-mouse IgG as the enzyme-labeled antibody. This established method was used to detect the antibody dynamic regularity of goats which are artificially immunized *Mmm* and was also compared with a commercial ELISA kit. Further, the sera of 1011 different cattle from border provinces of China were monitored using a candidate Mab 3A8 cELISA. The detection results of known background sera used in this study indicate that a candidate diagnostic marker was successfully identified by analyzing all the coding proteins of *Mmm* in this research, and the cELISA established based on the Mab 3A8 against this protein can detect CBPP-positive serum with specificity and has no cross-reaction with other related epidemic disease-positive sera. In addition, we tested the sera collected from the border areas of China using the established ELISA, and no positive sample was detected. The research protocol of the CBPP cELISA established in this study is different from the traditional method, which can greatly reduce the investment of manpower and capital and save development time. We believe that this study’s protocol could serve as a reference for the development of detection methods for mycoplasma and other complex pathogens.

**Key points:**

• *A Mmm-specific diagnostic marker was obtained based on protein characteristics.*

• *A cELISA was established for CBPP serum antibody detection.*

• *The serological investigation was conducted for CBPP in the border areas of China.*

**Supplementary Information:**

The online version contains supplementary material available at 10.1007/s00253-024-13127-0.

## Introduction

Contagious bovine pleuropneumonia (CBPP) is a bovine disease caused by *Mycoplasma mycoides* subsp. *mycoides* (*Mmm*); the susceptible animals are mainly cattle, but there are also reports of *Mmm* isolated from sheep and goats (Srivastava et al. 2000; Alhaji et al. 2020). The incubation period of CBPP varies from 3 weeks to 6 months. According to clinical manifestations, this disease can present in three forms: acute, subacute, and chronic. The infected cattle mainly present expiratory dyspnea, fever, runny nose, anorexia, etc. In severe cases, the infected cattle can be suffocated to death. When CBPP first appears in a herd, the mortality may exceed 50%, and it can reach 90% during the outbreak (Dudek et al. 2021; Di Teodoro et al. 2020). However, subacute and chronic forms are common and result in subclinical infections which may be the cause of the continuing spread of the disease. The report on CBPP can be traced back to Europe in the sixteenth century (Di Teodoro et al. 2020). At that time, the disease was confined to the Alps and Pyrenees. In the nineteenth century, it spread rapidly across the European continent through the Netherlands and Switzerland. In 1850, it was introduced into Scandinavia, the USA, and Australia through the UK. In the late nineteenth century, it penetrated into New Zealand, India, China, Mongolia, Korea, and Japan through Australia (ter Laak 1992). At the beginning of the twentieth century, many countries eliminated CBPP through culling or post-immunization stamp-out strategies. At present, CBPP is still causing epidemics in Sub-Saharan Africa. It is reported that the direct and indirect economic losses caused by CBPP are as high as 44.8 million euros (€ 44.8 million) per year in Africa (Tambi et al. 2006). CBPP arrived in China with the introduction of dairy cows in 1919, and the last infected animal was culled in 1989, and then CBPP never emerged in this country again. In 2011, China passed the WOAH certification and became a non-CBPP country (Xin et al. 2012).

Accurate and rapid diagnosis is very important to successfully control the prevalence of CBPP. Researchers have been making unremitting efforts in the field of CBPP detection methods. In terms of pathogen detection, in addition to pathogen isolation and identification, the polymerase chain reaction (PCR) test has become an important means to identify *Mmm*. At present, many traditional PCR (Bashiruddin et al. 1999, 1994; Le Grand et al. 2004) and real-time PCR detection methods (Gorton et al. 2005; Lorenzon et al. 2008; Schnee et al. 2011) have been developed. Lots of serological diagnostic techniques have been described, such as complex fixation test (CFT) (Amanfu et al. 2000), latex agglutination (March et al. 2003), slide agglutination test (Turner et al. 1963), immunoblotting (IB), competitive ELISA, indirect ELISA (Lutta et al. 2018), and immunohistochemical (IHC) (Luciani et al. 2016). IB, CFT, and cELISA are the test methods recommended by WOAH. The sensitivity and specificity of CFT were 63.8% and 98% respectively, while the sensitivity of cELISA was equivalent to that of CFT, and the specificity can reach 99.9% (Le Goff et al. 1998). Unfortunately, so far, there is no single test that can detect all clinical stages.

As China is a CBPP-free country, it is illegal to manipulate live *Mmm* in conventional laboratories. In this study, a diagnostic marker was determined by comprehensive analysis of all the coding protein sequences of *Mmm*, and the monoclonal antibody (Mab) was prepared using this protein as an immunogen, and then a CBPP competitive ELISA (cELISA) was established. Further, more than 1000 bovine sera from some border regions of China were monitored by this method.

## Materials and methods

### Sera

The CBPP negative serum, positive serum of CBPP, *Mycoplasma bovis*, *Mycoplasma bovirhinis*, *Mycoplasma agalactiae*, *Escherichia coli*, infectious bovine rhinotracheitis virus, and *Mmm* immunized sheep sera were stored in our laboratory. The positive serum of bovine viral diarrhea virus, *bovine tuberculosis*, and *bovine paratuberculosis* were donated by the relevant teams of Harbin Veterinary Research Institute. Local sera were collected from Heilongjiang, Inner Mongolia, Guangxi, and Yunnan provinces of China.

### Screening of diagnostic marker

Because the fact that *Mmm* is a class I pathogenic microorganism regulated by China, it is not possible to handle live *Mmm* in general laboratories in this country. To screen candidate diagnostic markers, this study obtained all protein sequences of *Mmm* strain PG1 from the NCBI database; the transmembrane region analysis software DeepTMHMM (DTU/DeepTMHMM – BioLib) (Alhaji et al. 2020) was employed to screen transmembrane proteins, and then the proteins that have an extracellular region with more than 100 amino acids and contain at least two transmembrane regions were selected. Then, the NCBI Protein BLAST program (https://blast.ncbi.nlm.nih.gov/Blast.cgi?PROGRAM=blastp&PAGE_TYPE=BlastSearch&LINK_LOC=blasthome) was used to select the protein regions that are conservative within *Mmm* and specific to other pathogens.

### Expression and purification of candidate proteins

The coding gene of the candidate marker was synthesized according to the protein sequence and cloned into pGEX-4 T-1 vector (GE) and pMAL-c5X vector (NEB), respectively, and then expressed and purified according to the manufacturer’s instructions. The gene of the extracellular region which contains the candidate marker was synthesized and cloned into the pGEX-4 T-1 vector, expressed, and purified as mentioned above.

### Antigenicity verification of candidate protein

The diagnostic marker expressed in pGEX-4 T-1 was tested by dot-ELISA with CBPP-immunized goat sera to confirm that the selected protein can react with CBPP positive sera and the negative serum as control.

### Production and identification of Mab

Monoclonal antibodies were prepared according to the method established by Köhler and Milstein (Köhler et al. 1975). Briefly, five 6-week-old female BALB/c mice (supplied by the Laboratory Animal Center of the Harbin Veterinary Research Institute, CAAS) were immunized subcutaneously with 50 μg of recombinant maltose-binding protein (MBP) labeled protein mixed with Freund’s complete adjuvant (Sigma-Aldrich). The booster vaccines consisting of 50 μg purified MBP labeled protein in an equal volume of Freund’s incomplete adjuvant were administrated 2 and 4 weeks after the primary immunization. The enhanced immunization was performed after the purified MBP labeled protein was mixed with Freund’s incomplete adjuvant in equal volume; each mouse was immunized subcutaneously with 50 μg of recombinant protein.

After the second booster vaccination, blood was collected from the tail end and serum was separated; then, the serum titer was evaluated by ELISA as previously described (Xu et al. 2015). Two days after the final booster, the feeder layer cells were prepared from the abdominal cavity cells of non-immunized mice 1 day before the fusion. The next day, the immunized mice were euthanized, and the spleen cells fused with SP2/0 myeloma cells at a ratio of 5–10:1 using polyethylene glycol (PEG 4000; Sigma Aldrich). Hybridoma cells were resuspended in HAT medium (containing 20% FBS, 100 μg/mL streptomycin, 100 IU/L penicillin, 100 mM hypoxanthine, 16 mM thymidine, and 400 mM aminopterin) and seeded into a 96-well plate. On the 7th day after fusion, the medium was removed and replaced with fresh HT medium (containing 20% FBS, 100 μg/mL streptomycin, 100 IU/L penicillin, 100 mM hypoxanthine, and 16 mM thymidine). Following HAT/HT selection, the positive cells were subcloned for 3 rounds with limited dilution and screened for the production of immunogen-reactive antibodies by indirect ELISA.

### Indirect ELISA

Indirect ELISA was used to determine the titer of mouse serum and ascites according to the protocol described previously with a few modifications (Xu et al. 2015). Briefly, microplates were sensitized at 4 °C overnight with the affinity-purified GST label protein at 5 μg/mL. The sensitized plates were incubated with test culture supernatants from hybridoma cells at 37 °C for 1 h, with goat anti-mouse IgG (H + L) conjugated with horseradish peroxidase (HRP) secondary antibodies (Sigma) at a 1:5,000 dilution at 37 °C for 1 h, followed by color development upon addition of tetramethylbenzidine substrate solution (Sigma). Color development was terminated with 2 M H_2_SO_4_, and the absorbance value of OD_450nm_ was recorded.

### Preparation and titration of ascites

The 10-week-old female BALB/c mice were intraperitoneally injected with 0.5 mL of Freund’s incomplete adjuvant. One week later, 1 × 10^5^ hybridoma cells were injected into the abdominal cavity of mice. The ascites were collected when the abdomen of mice was swollen. The supernatant of ascites was 10 times continuous dilution from 1:1000 and then titrated with indirect ELISA. Unimmunized mice serum was set as a negative control. The highest dilution with OD_450nm_ value ≥ 0.2 and P/N value ≥ 2.1 was considered the titer of ascites.

### Monoclonal antibody subtype identification

The subtype of Mab was identified by the SBA Cloning System-HRP Kit (Southern Biotech) according to the manufacturer’s instructions. Briefly, add 0.1 mL of hybridoma supernatant to each well of GST label protein coating plate, and incubate for 1 h at room temperature. Dilute HRP-labeled detection antibodies (1:250), add 0.1 mL of these conjugates to appropriate wells of the plate, and incubate for 1 h at room temperature. Add 0.1 mL of ABTS substrate solution to each well of the plate. Read the optical density of each well at 405 nm after substrate addition.

### The competitive ELISA

Costar ELISA plates (Corning) were coated with 0.1 ml of protein (1 μg/ml) diluted in 0.05 M carbonate buffer (pH 9.6) by incubation overnight at 4 °C. Plates were washed three times with PBST, and a 100 μl mixture of serum and Mab was added to each well. Place the plate in a 37 ℃ incubator for 1 h, discard the reaction liquid, and wash it three times with PBST. Then, add goat anti-mouse IgG antibody conjugated with HRP (Sigma) diluted at 1:5000, and react at 37 ℃ for another hour. After a final wash step, TMB (Sigma) was added to enable colorimetric analysis. The reaction was stopped by adding 50 μL of 2 M H_2_SO_4_, and optical density (OD) was measured at 450 nm. For each sample tested, the value of percentage inhibition (PI%) is calculated using the following formula: PI% = 100 x (OD_Mab_—OD_Sample_)/ (OD_Mab_ – OD_Blank_). The cut-off value between positive and negative sera was calculated with GraphPad Prism based on the analysis of a collection of 32 known CBPP-positive and 614 CBPP-negative serum samples.

### Validation of the competitive ELISA

To determine the cross-reaction with the other relevant epidemic disease-positive serum, several positive sera were tested by the cELISA. These sera included the CBPP negative serum, positive serum of CBPP, *Mycoplasma bovis*, *Mycoplasma bovirhinis*, *Mycoplasma agalactiae*, *Escherichia coli*, *bovine tuberculosis*, *bovine paratuberculosis* infectious bovine rhinotracheitis virus, and bovine viral diarrhea virus, with one serum of each species. One hundred and four serum samples collected from four *Mmm* immunized goats were detected with the cELISA to analyze the seroconversion. To compare the cELISA established in this study with the CBPP commercial kit produced by IDEXX company, 52 bovine and 79 *Mmm* immunized sheep serum samples were tested using these two methods. The Kappa values were calculated based on the detection results for consistency analysis.

### Seroprevalence of CBPP antibodies in bovine sera from Inner Mongolia, Heilongjiang, Guangxi, and Yunnan provinces, China

With the successful establishment of the cELISA, we collected bovine sera from Inner Mongolia, Heilongjiang, Guangxi, and Yunnan for CBPP antibody detection. These areas are located on the border of China. The monitoring of bovine serum samples in these areas is of great significance to prevent CBPP from being reintroduced and maintain the state of no epidemic.

## Results

### Screening of diagnostic marker

A total of 1016 proteins of the *Mmm* strain PG1 were obtained from the NCBI database (GenBank: BX293980.2). With the analysis of the transmembrane region, it was found that there were 11 proteins with more than two transmembrane regions, and the extracellular region was composed of more than 100 amino acids and subcellular localization on the cell membrane (Table S1). With BLAST analysis, the extracellular domain of a hypothetical protein (GenBank: CAE77242.1), with an amino acid coding range of 72 to 105, was selected as a candidate diagnostic marker.

### Expression and purification of proteins

The coding gene of HyP_72-105_ was synthesized according to the amino acid sequence and inserted into the pGEX-4 T-1 and pMAL-c5X vector; the recombinant plasmids were designated as pGEX-HyP_72-105_ and pMAL-HyP_72-105_ respectively. In addition, the gene encoding the extracellular region of 29 to 166 was synthesized and cloned into the pGEX-4 T-1 vector and named pGEX-HyP_29-166_.

**Fig. 1:**
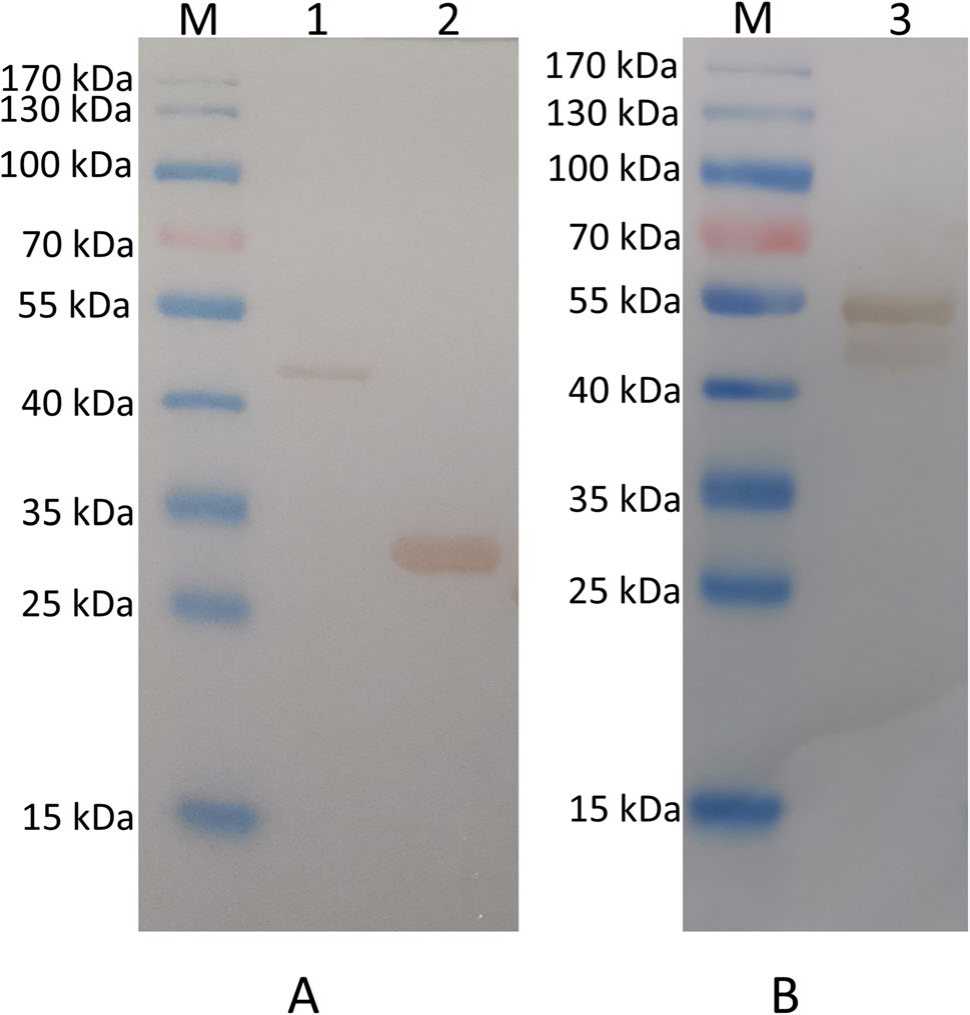
Prokaryotic expression and purification protein identification. The GST-HyP_29-166_ (**A**) and GST-HyP_72-105_ protein (**A**) were identified with anti-GST antibodies, and the MBP-HyP_72-105_ protein (**B**) was identified with anti-MBP antibodies. The three proteins were successfully expressed. Line M, protein marker; line 1, GST-HyP_29-166_; line 2, GST-HyP_72-105_; line 3, MBP-HyP_72-105_

Prokaryotic expression and recombinant protein purification of pMAL-HyP_72-105_, pGEX-HyP_72-105_, and pGEX-HyP_29-166_ were performed according to the procedures provided by NEB and GE, and then the HyP_72-105_ protein with MBP/GST tag and the HyP_29-166_ protein with GST label were obtained (Fig. 1). These proteins were designated as MBP- HyP_72-105_, GST-HyP_72-105_, and GST- HyP_29-166_.

### Antigenicity verification of GST-HyP_72-105_

**Fig. 2:**
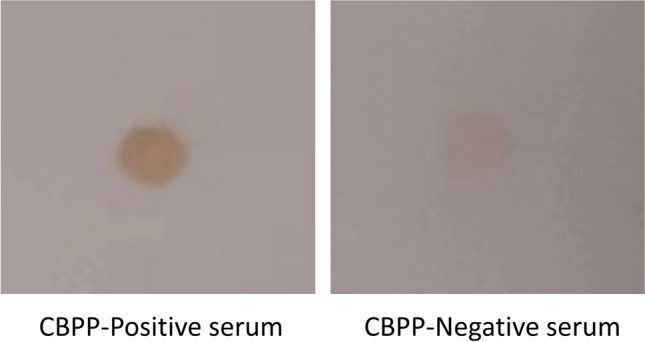
Identification of candidate protein reactivity to CBPP-positive serum. The antigenicity of GST-HyP_72-105_ was identified with sera from *Mmm* immunized and control goats. The GST-HyP_72-105_ had good reactivity with CBPP positive serum but not negative one

The antigenicity of GST- HyP_72-105_ protein was verified with CBPP-positive goat serum by dot-ELISA; the result showed that this protein could react with CBPP-positive serum but not CBPP-negative one (Fig. 2).

### Preparation and identification of monoclonal antibody

The BALB/c mice were immunized with protein MBP- HyP_72-105_, and the Mab was screened according to the protocol mentioned above. Finally, a hybridoma cell line stably secreting anti-HyP_72-105_ antibody was obtained, and it was named 3A8. The heavy chain of the Mab is IgG1, and the light chain is κ chain. The antibody titer in ascites was 10^5^.

### cELISA establishment for detection of serum antibodies against *Mmm*

With the purified GST-HyP_29-166_ protein as the coating antigen and Mab 3A8 as the detection antibody, a cELISA was established for CBPP positive serum detection. The optimal concentration of GST-HyP_29-166_ protein, 3A8 antibody, bovine serum to be tested, and goat anti-mouse IgG/HRP antibody was standardized by checkerboard titrations. The optimal concentration of the coated GST-HyP_29-166_ recombinant protein was 1 μg/mL, and the tested serum was 20 times diluted. The working concentration of ascites is 1:1250. The optimal dilution of goat anti-mouse IgG/HRP was 1:5000. A colorimetric readout was facilitated by the addition of 50 μl TMB. The OD of each well was read at 450 nm using a microplate reader.

**Fig. 3:**
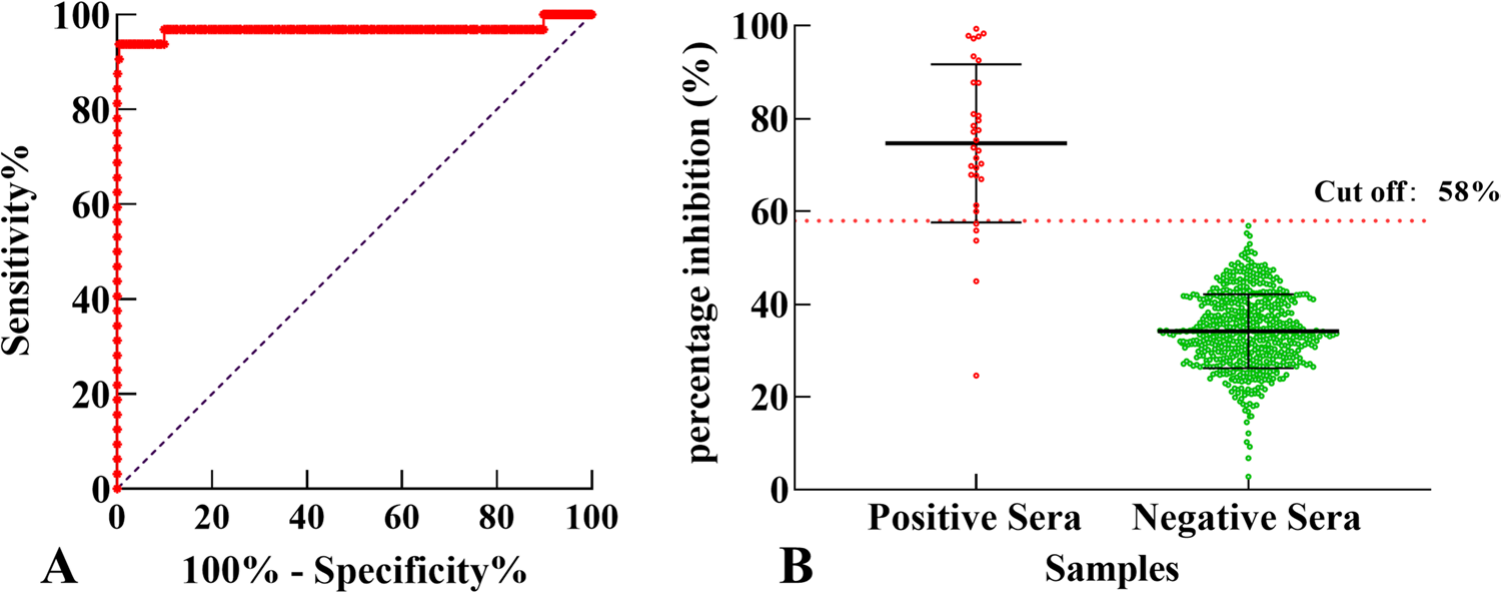
Determination of cELISA cut-off value. The established cELISA was used to detect 614 negative and 32 positive sera, and the optimal cut-off value was determined to be 58%. When the cut-off value was selected, the sensitivity of the cELISA was 84.38% (68.25% to 93.14%), and the specificity was 100% (99.38% to 100.0%) at 95% confidence. **A** The ROC curve of cELISA, with the *X*-axis and *Y*-axis representing the true-positive rate and false-positive rate of the detection method, respectively. **B** The distribution of negative and positive samples with a cut-off value of 58%, with negative and positive serum on the *X*-axis and blocking rate on the *Y*-axis

To determine the cut-off level of PI% for the cELISA, 32 CBPP positive sera and 614 CBPP negative sera were examined (Fig. 3). The PI% cut-off value was analyzed by GraphPad Prism statistical software, and a PI% of ≥ 58% was considered positive. At a PI% of < 58%, the serum antibody value was considered negative. With this cut-off value, the sensitivity of cELISA is 84.38% (68.25% to 93.14%), and the specificity is 100% (99.38% to 100.0%) at 95% confidence.

**Fig. 4:**
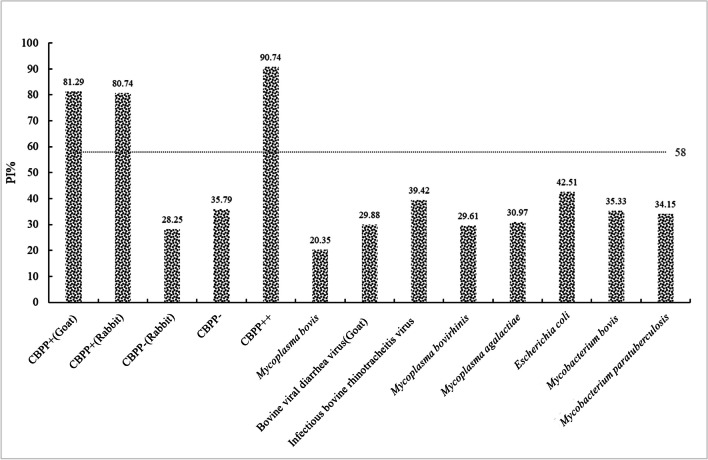
Cross-reactivity identification for CBPP cELISA. To determine the cross-reaction with the other relevant epidemic disease positive serum, several sera were tested by the cELISA. The PI% of the CBPP positive serum was more than 58%, while the PI% of the other sera was less than 58%. The cELISA can specifically detect the serum of animals infected with *Mmm* but has no cross-reaction with the sera of *Mmm*-free animals

To further verify the cross-reaction to the positive sera of other bovine diseases, the cELISA was used to simultaneously detect CBPP-positive serum, CBPP-negative serum, and positive sera of several bovine pathogens (Fig. 4). The PI% of the CBPP-positive serum was more than 58%, while the PI% of the other sera was less than 58%. This means that the cELISA established in this study can specifically detect the serum of animals infected with *Mmm* but has no cross-reaction with the serum of *Mmm*-free animals.

**Fig. 5:**
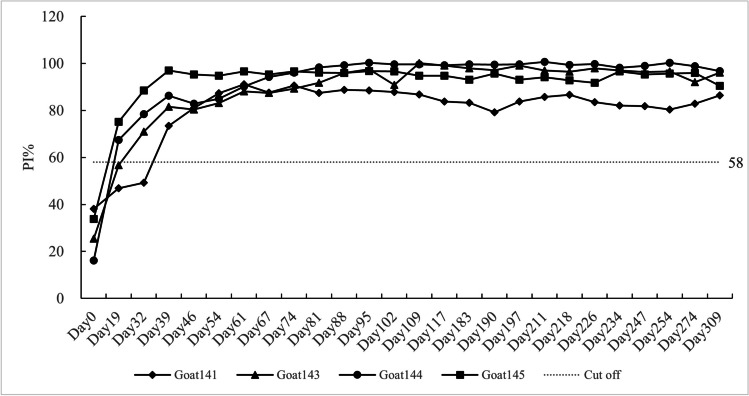
Detection of *Mmm*-immunized goat sera. The sera from 4 goats were tested with cELISA, serum positive conversion can be detected within 2 to 4 weeks, and the positive antibody lasts until 309 days after immunization

The ELISA established in this study was used to detect 104 serum samples; these sera were collected from 4 goats which have been immunized with inactivated *Mmm*. The seroconversion emerged 2 to 4 weeks after vaccination, and the HyP_72-105_ specific antibody of the immunized animals maintain at a high level until 309 days after immunization (Fig. 5).

We also evaluated the coincidence rate of the c-ELISA established in this study and a commercial cELISA kit (IDEXX) for CBPP detection. Seventy-nine samples of immunized goat serum and 52 samples of bovine serum were used to compare the two methods (Table 1). There are 55 negative and 72 positive sera were identified by both methods. However, 4 negative sera confirmed to cELISA were judged as positive by the IDEXX kit. The Kappa value of the two detection methods is 0.94, which indicates that the diagnosis results of the two methods are consistent.

**Table 1:** Correlation analysis of IDEXX and cELISA_3A8_

		IDEXX		
		Neg	Pos	Total
cELISA	Neg	55	4	59
	Pos	0	72	72
	Total	55	76	131

Kappa = 0.94

### Epidemiological survey for border samples

**Fig. 6:**
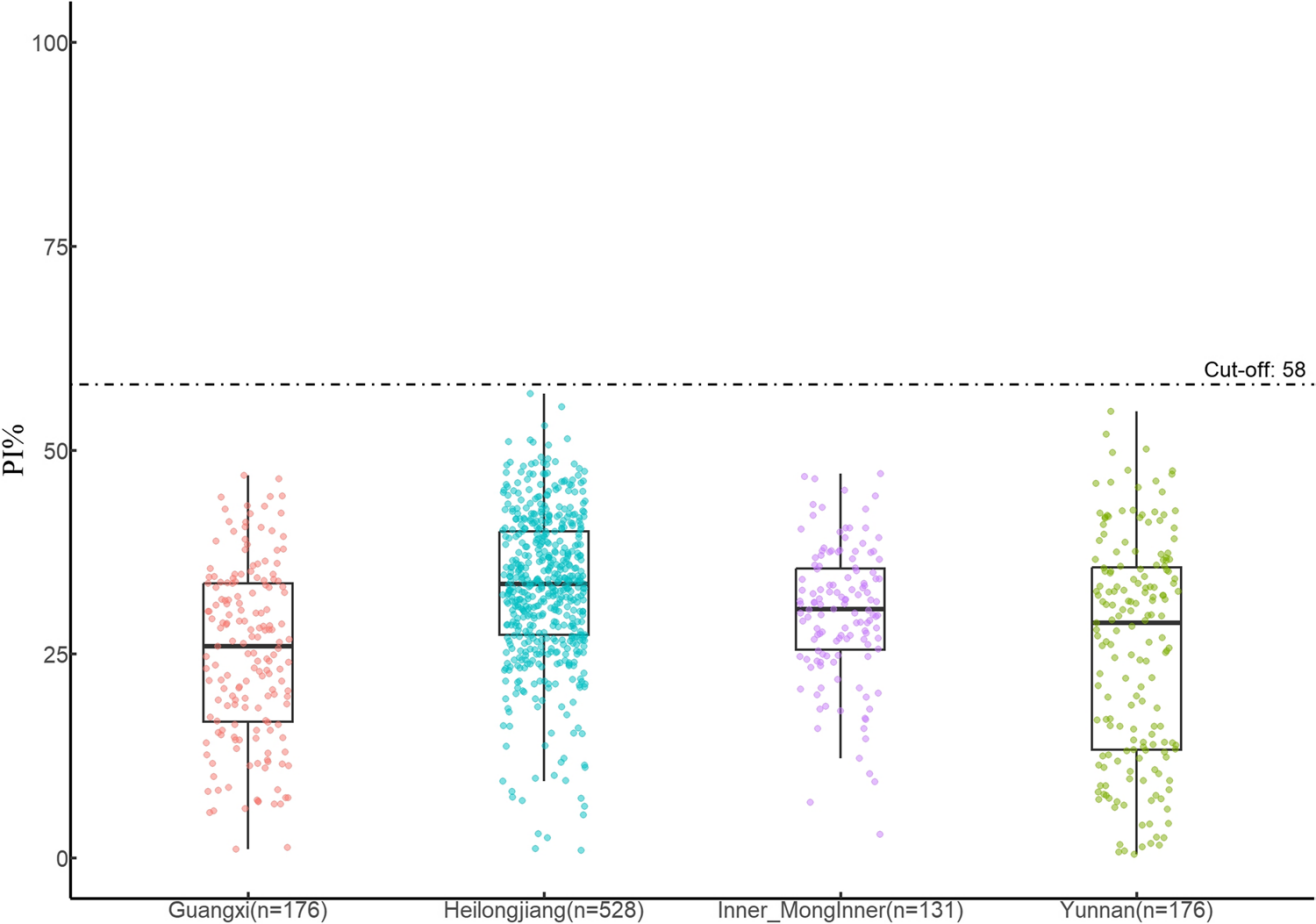
Serum detection of local samples. A total of 1011 bovine serum samples from Heilongjiang, Guangxi, Yunnan, and Inner Mongolia were tested by the cELISA established in this study, and all samples were negative for CBPP. The distribution of percentage inhibition for serum samples was displayed by the box plot

A total of 1011 bovine sera collected from Heilongjiang (528), Guangxi (176), Yunnan (176), and Inner Mongolia (131) in 2022 were examined by the cELISA established in this study. All of the samples were CBPP negative (Fig. 6).

## Discussion

In this study, we combined bioinformatics and traditional technologies to screen *Mmm*-specific protein, prepared an anti-*Mmm*-specific antibody based on this protein, and established a cELISA for CBPP antibody detection. *Mmm* is a complex pathogenic agent that encodes hundreds of proteins, and there are high-frequency mutations (Li et al. 2016; Westberg et al. 2004) in immunoreactive proteins due to immune escape and other reasons, which makes it very difficult to screen diagnostic markers of *Mmm*. Although a large number of studies have focused on the *Mmm* antigen protein selection, and there are also reports of various diagnostic methods, most of these studies are still stuck in the laboratory research stage, which indicates that traditional biological technology is not efficient in solving problems in the field of *Mmm* diagnosis (Alhaji et al. 2020; Di Teodoro et al. 2020).

The *Mmm* was prevalent in China and caused serious economic losses to animal husbandry several years ago (Xin et al. 2012). After the elimination of CBPP, China listed CBPP as a Class I animal disease, and its pathogen was ruled as a Class I pathogenic microorganism. In China, conventional laboratories have been prohibited from the operation and storage of live *Mmm*. In recent years, bioinformatics has developed vigorously. With the continuous progress of sequencing technology, the genome information of various pathogens has been increasingly improved (van Dijk et al. 2018). New biological algorithms have greatly improved the prediction ability of bioinformatics software. The emergence of online tools and the improvement of the computing power of supercomputers and personal computers have given everyone more opportunities to enjoy the benefits of information technology than before (Angarica et al. 2017). Mycoplasma membrane-associated proteins often have excellent immunogenicity and are closely involved in the colonization, replication, and pathogenicity of mycoplasma (Adamu et al. 2020; Archer 1981; Kostyal et al. 1994; Pan et al. 2022). The PG1 strain of *Mmm* was analyzed to screen the membrane protein, and an extracellular region of a transmembrane protein, which has diagnostic potential, was successfully identified. We also verified the antigenicity of this protein segment with CBPP-positive serum. On the premise of confirming its antigenicity, a Mab specific recognized this protein segment was prepared, and a cELISA was successfully established to detect the serum antibody of *Mmm*-infected animals. This means that it is feasible to combine bioinformatics with traditional biology to develop serological diagnostic methods. In particular, it has a potential application prospect for pathogens that encode a large number of proteins, have serious cross-reactions, or cannot carry out live-bacteria operations. Of course, this research is just a preliminary attempt, and the screening process is still relatively rough, only using conventional data and general algorithms and software. If more specific algorithms and databases were employed, the efficiency, reliability, and economy of screening will be greatly improved.

Pathogen isolation and identification is the most conventional laboratory method for CBPP diagnosis, and the serological test is usually considered a herd-level detection method. For the ELISA method, both the specificity and sensitivity have to be considered equally when setting the cut-off value. However, the cELISA for CBPP is somewhat special. The cELISA of CBPP, as a herd-level serological test, has more stringent requirements for specificity, for the reasons that CBPP has been eradicated in many countries. For example, the cELISA method recommended by WOAH has a sensitivity of about 70% and a specificity of 99.8–99.9% (Le Goff et al. 1998). The ELISA established in this study also follows this principle. Since CBPP has been eliminated in China, available positive serum of this disease is very limited. However, China is a CBPP epidemic-free area certified by WOAH, so it is relatively easy to obtain CBPP-negative serum. Therefore, we determined the cut-off value of the cELISA in two ways: One is the method of adding 3 times of standard deviation to the average value of negative serum samples, and the other is to use GraphPad Prism software to make statistics on known negative and positive serum samples. The values obtained by these two methods are close, so only the result recommended by GraphPad Prism was employed. Due to the limitation of the number of positive sera, the sensitivity parameter evaluation of the cut-off value may be affected to some extent. We will revise it in the follow-up study. In this research, 131 serum samples were used to compare the cELISA established here with the commercial CBPP test kit produced by IDEXX company; the results showed that they had a high correlation.

CBPP was first introduced in China from 1918 to 1919. It was one of the major epizootics that endangered Chinese cattle breeding, and the degree of damage was only second to rinderpest (Xin et al. 2012). Between 1949 and 1989, 178,570 cattle died from CBPP, with an estimated loss of 356 million Chinese yuan (Xin et al. 2012). In 1956, to control this disease, a vaccine with high protection rate was developed based on the *Mmm* Ben-1 strain, and the duration of immunization could reach 28 months. Then, China eliminated CBPP through vaccination combined with a culling policy. No CBPP positive animals emerge again since 1989. In 2011, China obtained WOAH epidemic-free zone certification for CBPP (Xin et al. 2007, 2012). Since then, China has conducted a CBPP monitoring program according to the requirements of WOAH every year. Unfortunately, the prevalence of CBPP in China’s surrounding countries is not very clear, and it is necessary to increase the level of epidemiological investigation in border areas. However, there is no cELISA or other high-throughput serological detection kit produced by China at present. The promotion of the cELISA established in this study will help improve the monitoring ability of CBPP in China.

## Supplementary Information

Below is the link to the electronic supplementary material.

Supplementary file1 (PDF 38 KB)

## Data Availability

All data supporting the findings of this study are available from the corresponding author on reasonable request.
