# Supplementary material for: A candidate competitive ELISA based on monoclonal antibody 3A8 for diagnosis of contagious bovine pleuropneumonia
Source: Appl Microbiol Biotechnol. 2024 Apr 8;108(1):290. doi: 10.1007/s00253-024-13127-0 (PMC11001708; doi:10.1007/s00253-024-13127-0)
Supplement: Supplementary file 1 — Supplementary file1 (PDF 38 KB) [file 253_2024_13127_MOESM1_ESM.pdf]

Applied Microbiology and Biotechnology

A candidate competitive ELISA based on monoclonal antibody 3A8 for diagnosis of contagious bovine pleuropneumonia

Qi Wu[1], Zhixin Ma[1], Qiao Pan[1], Tong Liu[1], Yidan Zhang[3], Jiuqing Xin[1*], Qingyuan Xu[1,2*]

1. State Key Laboratory for Animal Disease Control and Prevention, Harbin Veterinary Research Institute, Chinese Academy of Agricultural Sciences, Harbin, China.

2. Institute of Western Agriculture, Chinese Academy of Agricultural sciences, Xinjiang, China.

3. Shanghai Veterinary Research Institute, Chinese Academy of Agricultural Sciences.

*Corresponding authors: Jiuqing Xin, Qingyuan Xu

Tel: +86-451-51051799; Fax: +86-451-51997166

E-mail address: xuqingyuan@caas.cn

Table S1 Proteins identified by bioinformatics analysis

Accession Number	Molecular Weight(kDa)	Length(aa)	Number of Transmembrane Helix	Outside Region
CAE76658.1	42.6	363	4	56-296
CAE76707.1	64.5	581	8	212-341
CAE76727.1	50.1	432	9	82-218
CAE76825.1	46.3	414	6	90-203
CAE76869.1	120	1042	7	588-1042
CAE76956.1	82	704	4	1-478
CAE76964.1	70	610	6	191-583
CAE77242.1	87	770	2	29-707
CAE77101.1	101	873	11	370-574
CAE77527.1	64	588	10	47-155
CAE77529.1	57	487	5	187-487
